# Should We Embed in Chemistry? A Comparison of Unsupervised Transfer Learning with PCA, UMAP, and VAE on Molecular Fingerprints

**DOI:** 10.3390/ph14080758

**Published:** 2021-08-02

**Authors:** Mario Lovrić, Tomislav Đuričić, Han T. N. Tran, Hussain Hussain, Emanuel Lacić, Morten A. Rasmussen, Roman Kern

**Affiliations:** 1 Know-Center, Inffeldgasse 13, 8010 Graz, Austria; mlovric@know-center.at (M.L.); tduricic@tugraz.at (T.Đ.); htran@know-center.at (H.T.N.T.); hhussain@know-center.at (H.H.); elacic@know-center.at (E.L.); 2 Centre for Applied Bioanthropology, Institute for Anthropological Research, 10000 Zagreb, Croatia; 3 Institute of Interactive Systems and Data Science, Graz University of Technology, Inffeldgasse 16C, 8010 Graz, Austria; 4 Copenhagen Studies on Asthma in Childhood, Herlev-Gentofte Hospital, University of Copenhagen, Ledreborg Alle 34, 2820 Gentofte, Denmark; morten.arendt@dbac.dk; 5 Department of Food Science, University of Copenhagen, Rolighedsvej 26, 1958 Frederiksberg, Denmark

**Keywords:** manifold learning, machine learning, rdkit, embeddings, Tox21, principal component analysis, autoencoder

## Abstract

Methods for dimensionality reduction are showing significant contributions to knowledge generation in high-dimensional modeling scenarios throughout many disciplines. By achieving a lower dimensional representation (also called embedding), fewer computing resources are needed in downstream machine learning tasks, thus leading to a faster training time, lower complexity, and statistical flexibility. In this work, we investigate the utility of three prominent unsupervised embedding techniques (principal component analysis—PCA, uniform manifold approximation and projection—UMAP, and variational autoencoders—VAEs) for solving classification tasks in the domain of toxicology. To this end, we compare these embedding techniques against a set of molecular fingerprint-based models that do not utilize additional pre-preprocessing of features. Inspired by the success of transfer learning in several fields, we further study the performance of embedders when trained on an external dataset of chemical compounds. To gain a better understanding of their characteristics, we evaluate the embedders with different embedding dimensionalities, and with different sizes of the external dataset. Our findings show that the recently popularized UMAP approach can be utilized alongside known techniques such as PCA and VAE as a pre-compression technique in the toxicology domain. Nevertheless, the generative model of VAE shows an advantage in pre-compressing the data with respect to classification accuracy.

## 1. Introduction

Chemical (or molecular) representation is an important topic in cheminformatics [1] and quantitative structure–activity relationships (QSARs), as QSAR model quality depends largely on the predictive features defined by the task at hand, i.e., mapping a feature space (X) onto a target chemical or biological activity (y). Besides using molecular descriptors, which are numerous and sometimes hard to explain [2], the development of deep learning [3,4,5] and big data [6,7,8,9] gave rise to the utilization of various representations such as molecular fingerprints [10] and NLP-based methods like Mol2Vec [11]. With the advent of graph neural networks, researchers also started to learn from molecular images themselves [12]. More and more researchers are utilizing diverse representations to compare and find suitable features for solving the modeling problem [13,14,15,16] as no single feature set appears to be the optimal one. Hence, sometimes, combinations of features are also utilized [16]. Another difficulty in machine learning (and consequently cheminformatics) is the so-called curse of dimensionality, a term coined by Richard Bellman [17], which refers to various problems that arise when working with high-dimensional data (such as fingerprints) including increased chances of overfitting and spurious results. With high dimensionality, distances and densities (such as neighborhoods) might no longer be representative [18]. A well-established strategy, besides feature selection, to cope with this issue is dimensionality reduction, i.e., transforming the data into a low-dimensional space such that the resulting low-dimensional representation preserves certain properties of the original data. Such an approach has proven to be highly useful for numerous downstream machine learning tasks like classification [19], anomaly detection [20], and recommender systems [21]. Generally speaking, algorithms for dimensionality reduction can be categorized into three main groups, namely, matrix factorization, neighbor graphs, and deep learning-based approaches. Matrix factorization algorithms include approaches such as latent Dirichlet allocation [22], non-negative matrix factorization [23], and linear autoencoders [24]. However, the most commonly used approach is principal component analysis (PCA) [25]. It is founded on eigenvalue and eigenvector decomposition of symmetric semipositive definite matrices, and hence constitutes a clean linear orthogonal basis transformation to maximize variance explanation of both samples and variables. Indeed, the derived low-dimensional space—often referred to as scores—is a linear mapping of the original features. However, the systematic part of the original data is furthermore a linear mapping of the scores. In this way, PCA is a mathematically transparent and chemically interpretable tool for mapping data to a low-dimensional space and translating between this space and the original feature space. The linearity of PCA is what makes the method mathematically more concise than some nonlinear methods, but at the price of variance maximization as well as the inability to capture nonlinear phenomena in single dimensions. When it comes to nonlinear methods for dimensionality reduction, there are a number of noteworthy approaches, such as locally linear embedding [26], Laplacian eigenmaps [27], or t-SNE [26]. One of the most effective and commonly used methods that also falls into this category is uniform manifold approximation and projection for dimension reduction or UMAP [28]. It is a nonlinear method that works by utilizing local manifold approximations and combining their local fuzzy simplicial set representations in order to create a topological representation of the high-dimensional data. It then minimizes the cross-entropy between topological representations, thus optimizing the layout of the data representation. UMAP was employed already in understanding patterns in chemical/structural [29,30] and biological data [31,32] by transferring them to a lower dimensional space. More recently however, the category of nonlinear dimensionality reduction approaches has been extended with deep learning-based algorithms. The most prominent approach here is the traditional autoencoder model [33]. Autoencoders are (typically nonlinear) neural network architectures that learn to copy their input onto their output by passing the input through an intermediate bottleneck layer. As they were originally proposed, there has been a number of adaptations of the original autoencoder with variational autoencoders or VAEs [34] as one of the latest state-of-the-art methods. Autoencoders are often applied in cheminformatics for tasks like chemical space navigation and de novo molecular generation [4,29,35] or prediction [36]. The aim of this work is thus to evaluate chemical space information generated from fingerprints and investigate the impact of generating embeddings in an unsupervised manner using prominent linear and nonlinear methods. That is, we specifically look into the utility of PCA, UMAP, and VAE (hereinafter embedders) as pre-compression techniques for solving classification tasks with 12 binary toxic outcomes.

## 2. Results and Discussion

### 2.1. Setting the Baseline

Before presenting the results of embeddings as predictive features, we present here the baseline models trained on raw fingerprints. The results are shown in Table 1. Furthermore, we compared the results to recent work [37] where the results are also presented by means of MCC and trained on fingerprints (like in the present study). The limitations of this comparison are as follows: (a) the train–test splits are not the same; (b) there is a difference in fingerprint parameters; (c) we limited hyperparameter optimization, as our aim was to compare the results of the embeddings; (d) we used average values of the predictions on the test set for several random states; and (e) Zhang et al. use a Bayesian optimization instead of the grid-search we used. We also want to note that the classification results from Zhang et al. [37] show higher MCC values than the results in this work. Besides the mentioned differences, in our work, instead of using nested-CV, we report on a true external test set. The purpose of this comparison is to present the expectations towards the individual labels in Tox21.

### 2.2. Embedding Chemical Spaces

**Figure 1:**
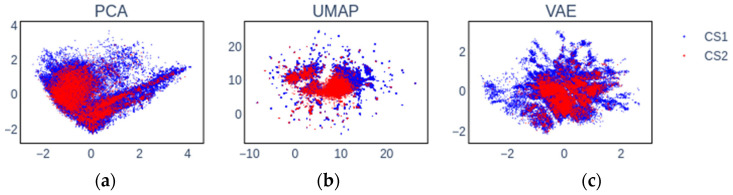
Exemplary visualization of CS1 (red) and CS2 (blue) show in 2D embedded space generated from molecular fingerprints by means of (**a**) principal component analysis (PCA), (**b**) uniform manifold approximation and projection (UMAP), and (**c**) variational autoencoders (VAEs).

In this section, the classification results of models trained on embeddings (internal/external) are presented and discussed. For the purpose of training the models, the fingerprints were subjected to transformations into low-dimensional spaces by either PCA, UMAP, or VAE. Visual examples of the transformations (CS1 and CS2) are shown in Figure 1 for both data sets.

Our first observation by visually inspecting Figure 1 is that the three studied embedding algorithms produce greatly different data representations owing to the underlying differences in how they operate. Furthermore, we observe that CS1 covers a larger chemical space in comparison with CS2 owing to the difference in data set size. Additionally, some areas of CS1 data space are barely covered in CS2, making it harder to transfer knowledge for these compounds. This behavior appears more pronounced in UMAP and VAE data representations. Finally, UMAP and VAE appear to produce a number of smaller observable clusters in comparison with PCA. This is an inherent consequence of UMAP, where indeed the underlying graphs of the samples are pruned, while PCA is a linear mapping, making it impossible to introduce discrete clusters if they are not directly present in the raw data.

### 2.3. Impact of Embedding Size and Information Content

**Figure 2:**
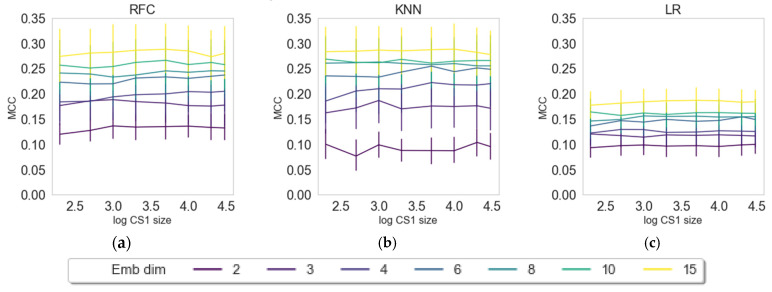
Dependence of classification results or transferred embeddings of CS2 by means of Matthews correlation coefficient (MCC) on the log-size of CS1 and dimensions of the PCA embeddings. The three figures represent three classifying algorithms, namely, (**a**) RFC, (**b**) KNN, and (**c**) LR.

**Figure 3:**
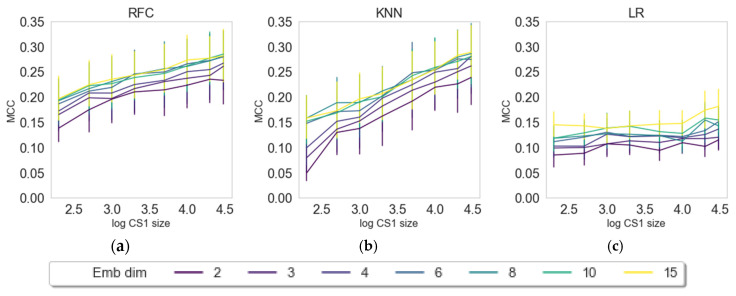
Dependence of classification results or transferred embeddings of CS2 by means of MCC on the log-size of CS1 and dimensions of the UMAP embeddings. The three figures represent three classifying algorithms, namely, (**a**) RFC, (**b**) KNN, and (**c**) LR.

**Figure 4:**
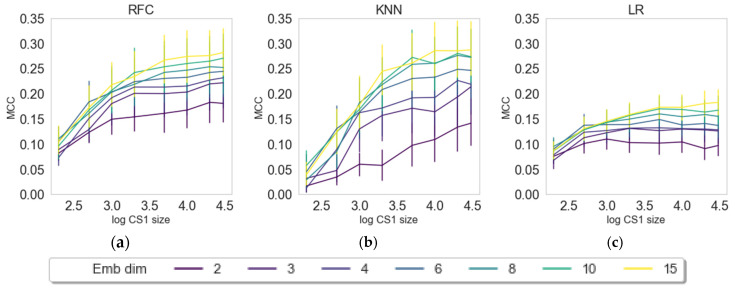
Dependence of classification results or transferred embeddings of CS2 by means of MCC on the log-size of CS1 and dimensions of the VAE embeddings. The three figures represent three classifying algorithms, namely, (**a**) RFC, (**b**) KNN, and (**c**) LR.

In this section, we want to evaluate the contribution of the number of latent variables (dimensions) and the data input size for creating embeddings from the external data set prior to solving the classification tasks (see Figure 2). The embedding approaches were varied in input size (200–30,000 compounds from CS1) and number of dimensions (2–15 latent variables) and evaluated for three random states, three different classifiers (RFC, KNN, LR), and three embedders (PCA, UMAP, VAE) as described in the Materials and Methods section. The results are depicted in Figure 2, Figure 3 and Figure 4.

The results for PCA (Figure 2) show on average an increase in the predictive quality of the models with a growing number of dimensions (principal components). The effect of information content by means of CS1 size is shown to be negligible as the MCC score remains steady for all different sizes of CS1.

Models embedded with UMAP (Figure 3) show distinctively different patterns compared with PCA. For KNN and RFC, there is a clear trend for increasing dimensions and information content, while there seems to be some randomness in LR with an existing trend for model improvement when increasing dimensions and information content. The third embedding algorithm, VAE, shows similar patterns to UMAP (see Figure 4). An observable difference to UMAP is that, for RFC and KNN, it seems to approach a plateau, while UMAP shows a steady increase. All machine learning methods show a clear increase of model quality with an increase of dimensions and CS1 data size.

PCA by definition only extracts linear features, and the ability to capture nonlinear behavior relies on the upstream use of nonlinear classifiers. Here, we indeed see that nonlinear phenomena are captured using kNN or RFC in contrast to logistic regression. On the other hand, PCA is powerful in filtering off stochastic white noise, simply because of the nature of white noise where there is no dependency across features. Indeed, this phenomenon is observed for the PCA embedding as the performance is insensitive towards the size of training data as well as classifier type, simply pointing out that the principal components’ directions are well defined even for small data set. It is interesting to observe that, with UMAP, we see a saturation regarding the number of dimensions (6, 8, 10, and 15 perform similarly), while with VAE, we observe the effect, but to a lesser extent. VAE seems to benefit more from input data size in comparison with UMAP, which we attribute to deep learning methods typically requiring larger data sizes for learning [38]. In further evaluations, we compared only the results on maximum dimension size (15) and maximum set size (30,000 compounds in CS1).

### 2.4. Internal versus External Knowledge

To evaluate whether the external knowledge (from CS1) is beneficial for the classification task in CS2, we list the MCC score of each embedding algorithm, trained (a) on external data from CS1 and (b) on internal data from CS2, in Table 2. One has to keep in mind that CS1 size was varied, while CS2 is fixed.

Our results have a few important takeaways. In comparison with PCA, both UMAP and VAE were able to achieve better results overall when fitted on external knowledge versus fitting on internal knowledge. With external knowledge, PCA achieved better results on two labels and equal results (rounded on two decimals) on three. UMAP and VAE performed similarly as both approaches performed better when trained on external knowledge on five labels and performed equally to respective models trained on internal knowledge on five labels (out of 12 labels in total). It is important to note that, even though UMAP and VAE showed similar performance overall, they performed differently for different labels. For example, UMAP showed a strong improvement in MCC score for NR-AR-LBD when using external knowledge, whereas VAE did not improve in comparison with VAE trained on internal knowledge. There was only one label (SR-ARE) for which all three embedding algorithms yielded better results when trained on external knowledge.

### 2.5. Should We Embed? Does Embedding Win over Baseline?

**Figure 5:**
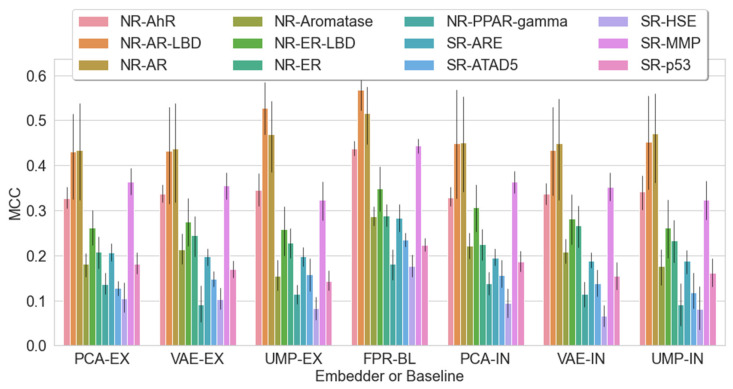
Comparison of machine learning classifications mean + error bar across all feature sets: FPR-BL = fingerprint baseline, PCA = principal component analysis (EX = external, IN = internal), UMP = uniform manifold approximation and projection, VAE = variational autoencoders. Each bar present nine runs (three classifiers × three random states).

Figure 5 and Table 3 compare the MCC score of all embedders (internal and external) to the fingerprint baseline model (FPR-BL) on each classification task. From Figure 5, it is clear that the fingerprint baseline model performs better overall for most of the labels.

Table 3 shows that the embedders do not increase in the score in general. However, for the NR-ER label, VAE embedders show an increase in MCC score. To present embedding capabilities, we compared the maximum values per embedder in Table 3. Each value is the maximum of nine machine learning experiments (three classifiers × three random states). For easier comparison, we set the FPR-BL maximum per label to 100%. The results show that 3 out of 12 labels embedded features can reach or surpass the baseline, namely, NR-AR, NR-AR-LBD, and NR-ER. With PCA, both internally and externally, the maximum value never reached those of the baseline, while with externally trained UMAP, only one target performed on par with the baseline. With the variational autoencoder, the baseline was reached three times. This shows the dominance of VAE over other embedders given the constraints in our experiments. In conclusion, by embedding molecular fingerprints, we can obtain a comparable and sometimes improved classification accuracy with toxicological models compared with no embedding. The main advantage in applying embedding techniques on the molecular fingerprints in this way is a reduced model complexity, by utilizing smaller feature sets, without the need to sacrifice predictive accuracy.

### 2.6. Insights into Latent Representations

The embedders compress information in an unsupervised way, thus the resulting output is based on the efficacy of the utilized approach; underlying data; and, to an extent, how well the hyperparameters are tuned. Therefore, it is difficult to predict whether utilizing the same compression techniques would be beneficial for use cases that are different from our problem of predicting 12 toxic outcomes. To better understand how the classification tasks can profit from compression, we calculated silhouette coefficients on the calculated embeddings within the data sets, s (Equation (1)), in the latent space (for 2D and 3D latent spaces as well as using external embeddings):

(1) $$\begin{array}{c} s\left(i\right)=\frac{b\left(i\right)-a\left(i\right)}{\max\left\{a\left(i\right),\;b\left(i\right)\right\}},if\;\left|C_{i}\right|>1\\ s\left(i\right)=0,if\;\left|C_{i}\right|=0 \end{array}$$

**Figure 6:**
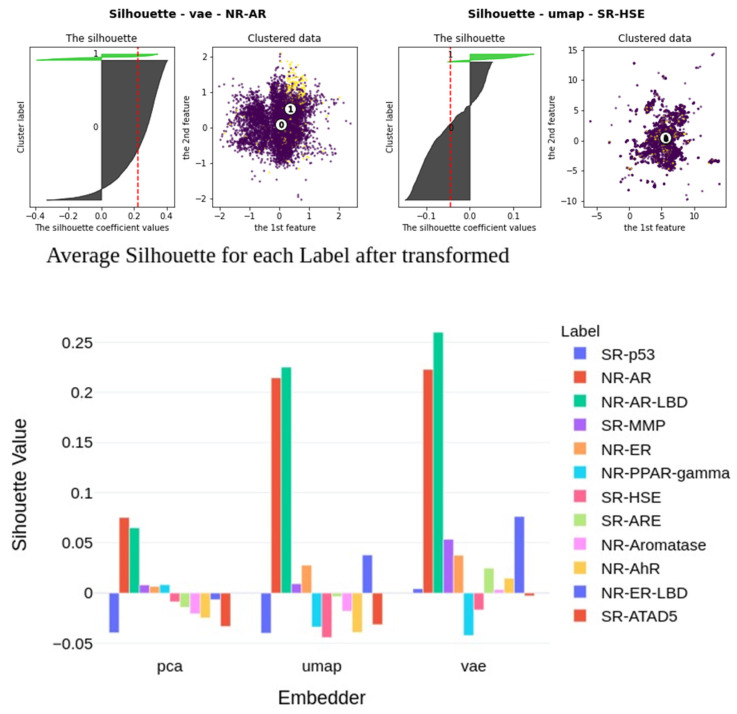
Top: Silhouette plots for best (top left) and worst (top right) performing clustering, which are VAE with NR-AR and UMAP with SR-HSE, respectively. The gray and green masses are points in two classes (0 and 1) with their silhouette coefficients, respectively. The dashed red line is the average value of both. The scatter plot visualizes their coordinates in the 2D embedded space. The number is their center points for each class (0 and 1). We notice that, for the best clustering, the silhouette coefficient tends to be higher, and points that belong to NR-AR label visually agglomerate together. Conversely, the silhouette coefficient seems to be negative for the worst clustering, and the points belonging to SR-HSE label are visually indistinguishable from other points. Silhouette coefficients for externally embedded CS2 data (bottom) by means of the three embedders (PCA, UMAP, VAE) calculated per label, which are presented by the color map. We see that VAE gains a higher silhouette coefficient on most tasks than UMAP or PCA, indicating a better separation.

In this equation, *a(i)* is the mean distance between a molecule *i* and all other molecules in the same cluster and *b(i)* is the mean distance of molecule *i* to all molecules in any other cluster. For each label, the coefficients are plotted in Figure 6. The average silhouette coefficients per label were correlated to the predictive quality (MCC), as shown in Table 4. Besides that, we compared the baseline models (raw fingerprints) and the imbalance ratio with respect to the MCC results of the classifiers that utilize embeddings. The results show that the predictive quality correlates almost perfectly (0.98+) with the baseline models, which means that achieving a good classification on raw data (i.e., fingerprints) will most likely also lead to good results after embedding.

Nevertheless, there is also a high correlation between the calculated silhouette coefficients and the predictive quality of embedded classifiers. This indicates that, even though the embedders distribute the classes without prior knowledge, they still seem to keep more relevant information regarding the given classification task. This turned out to be more relevant for the nonlinear methods (i.e., UMAP and VAE) than for PCA.

## 3. Materials and Methods

In this section, we present the data for conducting the study, the machine learning methods used for solving the classification tasks, the embedding techniques utilized, and the overall modeling pipeline.

### 3.1. Data

The data for the classification experiment (here named compound set 2 or CS2) were downloaded from the Tox21 public repository [39]. The chosen set is the 2014 Tox21 challenge subset with 12 toxicological endpoints related to stress response and nuclear receptor panels. This dataset was studied in many works and was subject to a plethora of reports on the outcomes [3,15,40,41]. Hence, it represents a baseline dataset for QSAR classification as it is imbalanced, chemically diverse, and large (~10k compounds), but has also several endpoints with different predictive capabilities (modeling challenges). Owing to the mentioned challenges this dataset offers, it has been subject to numerous studies in advanced machine learning methods [3,40], balancing methods [15,42], as well as novelties in chemical representation such as conformational resampling [41] and multitask learning [3]. We subjected the data to preprocessing as they consist of duplicated structures, which was reported previously [15]. During preprocessing, we removed structures that did not have valid SMILES or identifiers [7] and they were additionally converted to their canonical SMILES. Furthermore, we removed duplicates by both their IDs and SMILES. We removed inorganic compounds and metal-containing compounds as well as fragments. The procedure is inspired by [15,43] to keep the active part of the compounds. For the predictive tasks, Morgan fingerprints (FPR) were calculated for the 8314 structures by means of the RDKit library [44]. Owing to the possibility of colliding bits in fingerprints [45,46], we set the fingerprint vector length to 5120 bits and the radius to 2. In order to foster reproducibility, we made the scripts that are used for data preprocessing and feature engineering available already in our recent work [16].

The compounds used for generating external embeddings were retrieved from [47] and consist of 68,679 compounds. This compound set 1 (i.e., CS1) was preprocessed in the same manner as the Tox21 dataset described above. After preprocessing and duplicate removal, a total of 54,820 structures remained. During modeling, the structures present in both sets (CS1 and CS2) were removed from CS2 to avoid a target leak.

### 3.2. Machine Learning Methods

The task at hand is to predict the labels in Tox21, which are binary classes. For this, we employed three common classifiers, namely a random forests classifier (RFC) [48], logistic regression (LR) [49], and a k-nearest neighbor classifier (kNN) [50]. These algorithms are conventional tools when conducting machine learning studies and represent different inductive biases (e.g., assuming that the relationship between input attributes and the output of a LR algorithm is linear)). As the datasets are imbalanced, which makes them challenging when trying to avoid random classification issues [51], we employed penalization and optimization techniques to improve classification outcomes. In our experiments, we first randomly split the data into a train and test set with a 3:1 ratio (i.e., 75% of the data are part of the train set). To penalize the models for misclassification of the minor class (active compounds), we employed the Matthews correlation coefficient (MCC) [52] as a scoring function, as it was shown in our previous studies to work well for imbalanced sets [13,53]. MCC is defined by Equation (2), where TP, TN, FN, and FP are the elements of the confusion matrix given in Table 5.

(2) $$\mathrm{MCC}=\frac{TP\cdot TN-FP\cdot FN}{\sqrt{\left(TP+FP\right)\cdot \left(TP+FN\right)\cdot \left(TN+FP\right)\cdot \left(TN+FN\right)}}$$

The models were tuned with respect to their hyperparameters, which were found using exhaustive grid-search evaluated by cross-validation [54]. All models were trained with the scikit-learn library for Python [55].

### 3.3. Transfer Learning with Embeddings

#### 3.3.1. Principal Component Analysis (PCA)

PCA [25] is an algorithm for dimensionality reduction based on the maximization of variance in a lower-dimensional projected space. In that regard, PCA can be perceived as a linear autoencoder [56]. The mathematics of PCA is described in many textbooks, but in short, the original data (**X**~(*n,p*)) are represented by the product of two matrices, namely the scores (**T**~(*n,k*)) and the loadings (**P**~(*p,k*)), Equation (3):

(3) $$X=TP^{T}+E$$

where **E**~(*n,p*) is the residual matrix and *n, p,* and *k* are the number of samples, variables, and components, respectively. The parameters are estimated to capture as much of the variance in the original data in a least squares sense, and further to be orthogonal matrices, i.e.,

(4) $$\left\{T,P\right\}=argmax_{T,P}\left(\|X-TP^{T}{\|}_{2}^{2}\right)$$

**Figure 7:**
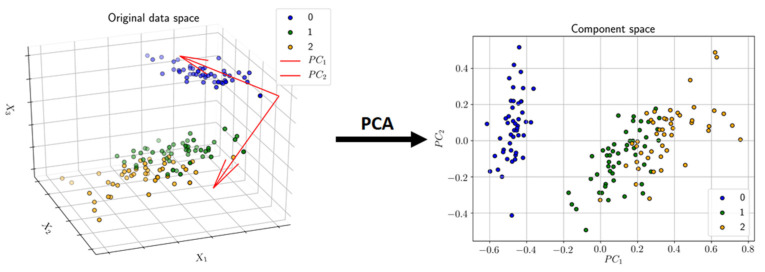
An example of dimensionality reduction by means of PCA. Instances/points in a 3D space (original space) are transformed into a 2D space of two latent variables called principal components (PC1 and PC2).

The combination of vectors of **T** and **P** are referred to as principal components, and used in various ways in, e.g., exploratory data analysis to map the multivariate sample distribution as well as interrogating feature2feature correlation structure, as well as—like in this work—to represent the data in a few meaningful features used for further analysis. A rewrite of Equation (4) above shows that the score space (**T**) is a linear mapping by the orthogonal basis represented by **P**: **T** = **XP**, and hence a rotation of the coordinate system as depicted in Figure 7.

#### 3.3.2. Uniform Manifold Approximation and Projection (UMAP)

The recent work of McInnes et al. [28] has tackled the problem of dimensionality reduction by generalizing linear approaches like PCA in order to be sensitive to a possible nonlinear structure in data. By applying a completely new field of mathematics, which is based on Riemannian geometry and algebraic topology, they developed the uniform manifold approximation and projection (UMAP) algorithm.

**Figure 8:**
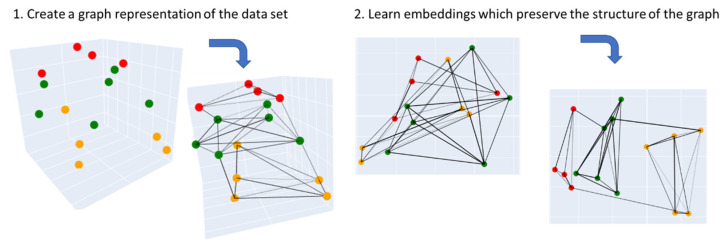
Visual explanation of how UMAP works. It first computes a graph representation of the input data, which is then used to learn embeddings that preserve the structure of the graph representation. Figures is redrawn based on ref. [57].

Using every available data point, UMAP first creates a graph with respect to the distances on the underlying topology and to the k-neighborhood of each element (as seen in Figure 8). The Laplacian eigenmaps dimensionality reduction method is then applied on that graph. The resulting graph is further modified by a forced directed graph layout algorithm, which minimizes the cross-entropy between this modified graph and the original one. In this manner, the resulting low-dimensional data representation is optimized to well preserve both the local and global structure of the original data. The main advantage of UMAP over PCA is that it is able to capture a more complex (nonlinear) structure in high-dimensional data, which is a desirable characteristic in our use-case. UMAP is able to achieve this by initially constructing a high-dimensional graph representation of the original data, followed by optimizing a low-dimensional graph to be as structurally similar to the original as possible. In this manner, the resulting low-dimensional data representation is able to well preserve both the local and global structure of the original data.

#### 3.3.3. Variational Autoencoders (VAE)

**Figure 9:**
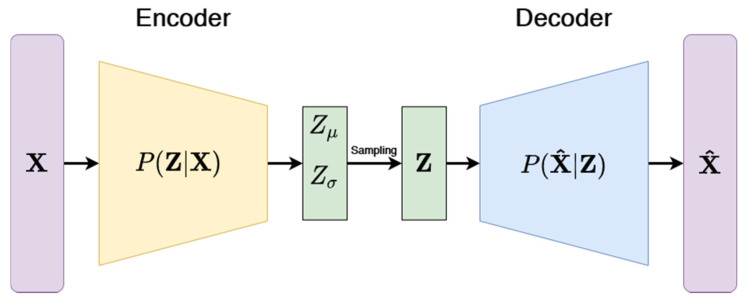
Architecture illustration of the variational autoencoder. Encoder compresses the input X into a latent representation Z. VAE is different to a standard autoencoder as it assumes that the input data have an underlying probability distribution (e.g., Gaussian) for which they try to optimize parameters. The decoder then attempts to reconstruct the original input from the representation by minimizing the reconstruction loss.

Since the work of Kramer [58], autoencoders have become a popular alternative to PCA in providing an effective method to reduce the dimensionality of data. This type of neural network is defined by a two-part architecture, which consists of an encoder and a decoder. In its simplest form, it has only one hidden layer (i.e., the information bottleneck), which is a low-dimensional representation of the original data. It is trained in an unsupervised manner to encode the data in a way that keeps the information loss minimal when the decoder attempts to recreate the input from the hidden layer. One popular extension of this approach is to use variational inference when extracting the latent representation [59]. The main difference lies in the fact that the network does not encode the input as a single point. Rather, it makes strong assumptions that the input data can be represented as a probability distribution like Gaussian and encodes the mean and variance of the data separately. As seen in Figure 9, the decoder of the variational autoencoder then samples the latent representation to produce a probability distribution of the low-dimensional representation. Such a probabilistic approach allows the variational autoencoder to be a generative model, i.e., the decoder is capable of creating completely new data that are similar to the observed data used for training the model.

#### 3.3.4. Embedder Training

**Figure 10:**
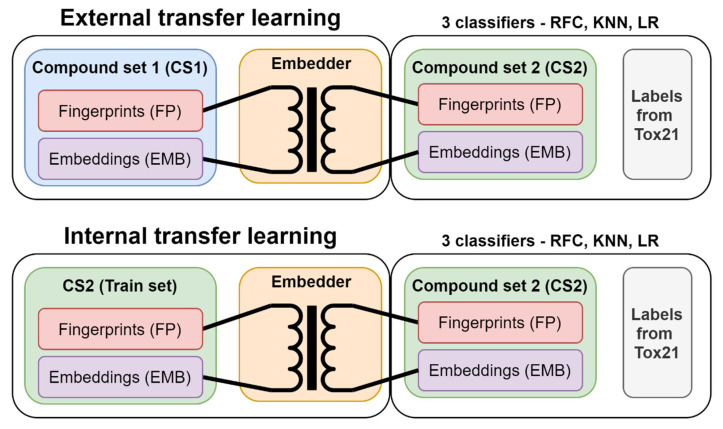
In external transfer learning, an embedder (PCA, UMAP, VAE) is fit on fingerprints on an external set of fingerprints (CS1). The same model (pre-trained embedder) is then utilized to encode fingerprints from CS2. In internal transfer learning, the embedder is fit on the pre-split train set of CS2 and used to encode the test set of CS2. The embeddings of CS2 were utilized for training predictive classification tasks.

**Figure 11:**
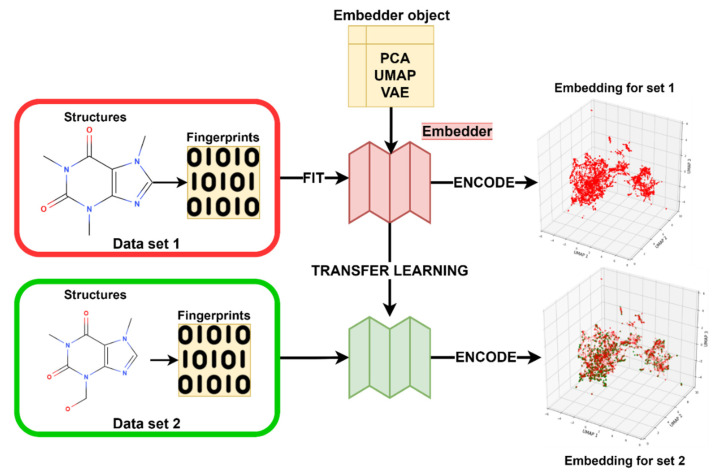
Schematics of chemical space transformation from fingerprint through a pre-trained embedder model. The transformation can either be conducted from an external data set to the data set of interest or within the data set of interest, but split into the train and test set.

The embedders are trained on molecular fingerprints. For this purpose, we created two sets of embedders: (1) embedders created with the external data set (CS1), which are then consecutively used to encode CS2, which results in the transformed representation of CS2 (hereinafter, external embeddings (EX)); and (2) embedders that were created on the respective pre-split train set of CS2 and used to encode the pre-split test set of CS2 (hereinafter, internal embeddings (IN)). Both procedures are shown in Figure 10. The two sets of embeddings were used for solving the classification task of the 12 toxicological labels alongside commonly used fingerprints. We have fitted the three embedding techniques (i.e., PCA, UMAP, VAE) on the fingerprints from the CS1 set in eight different data sizes of randomly selected compounds (200, 500, 1000, 2000, 5000, 10,000, 20,000, and 30,000 compounds) and embedding dimensions (i.e., number of latent variables: 2, 4, 6, 8, 10, and 15). The concept of how to train and apply embedders on chemical spaces is shown in Figure 11. The Tox21 dataset was transformed with each of the embedders subsequently and used in the machine learning prediction for each of the 12 toxicological endpoints. The full experimental matrix consists of 18,288 individual machine learning experiments (see Table 6).

#### 3.3.5. Modeling

A modeling pipeline was created and written in the programming language Python (v3.6.0). The pipeline is set as follows: **(1)** data for CS1 and CS2 are loaded, where CS2 involves the Tox21 modeling data (fingerprints—FPs, labels/endpoints) and CS2 fingerprints; **(2)** FPs columns for both CS1 and CS2 below 5% variance are removed; **(3)** removal of structures from CS2 which appear in CS1; **(4)** train and apply embedders; and **(5)** optimize classification models and apply them on embedded data, as shown in Table 6.

## 4. Limitations and Future Outlook

This study includes several limitations. First there are many other chemical representations besides fingerprints. Among those, interesting results may be revealed from approaches such as Mol2Vec [11] or graph-based methods [12]. The fingerprints parameters (5120-bit length and radius 3) do not have an optimal representation, but are rather based on suggestions from past research [46]. We limited also the embedded data set sizes owing to computational performance issues in training thousands of models. Therefore, with just a few latent variables uses compared with fingerprint, we might have experienced some information loss. Furthermore, there is a plethora of machine learning methods; here, we used only three that are well described and different by their paradigms in learning. The hyperparameters space for either machine learning or embedding algorithms can also be further explored for each individual method as well as the methods for choosing them, like Bayesian optimization [16,37]. These methods can also be applied to smaller labeled datasets, which is one of our aims in future research.

## 5. Conclusions

In this work, we evaluated the effects of pre-compression techniques on chemical space information generated from fingerprints and utilized in the domain of toxicology. Specifically, we focused on prominent linear and nonlinear techniques like PCA, UMAP, and VAE and showed their utility when using QSAR models, which are related to stress response and nuclear receptors. We showed that, with external knowledge that is transferred via a pre-trained embedder, we can classify toxicity outcomes with a reasonable model quality. The quality of the prediction, however, depends to a large extent on the class separation within each of the 12 toxic outcomes. The results of the silhouette coefficients suggest that nonlinear methods can achieve a much higher performance than PCA. Moreover, our research revealed that, for the utilized data sets, VAE exhibits much better results when compared with PCA and UMAP. Nevertheless, the recently popularized UMAP approach can still be employed for pre-compression as it shows the ability to maintain high-dimensional relationships.

## Figures and Tables

**Table 1:** A comparison of our baseline results trained on fingerprints to a similar study from Zhang et al. [37]. The results from Zhang are denoted with a “Z“, while the respective classifiers are as follows: L—lightGBM, R—random forests, S—support vector machines, X—XGBM, D—deep neural networks. The classifiers from this work are k-nearest neighbor classifier (KNN), logistic regression (LR), and random forests classifier (RFC), which are represented by their mean values per classifier, respectively. Additionally, the mean and max of all classifiers in this work are compared. The best baseline models in our work are marked with an superscript “a“, while the best models from Zhang are marked with an superscript “b“.

Label (endpoint)	Mean (all)	Max (all)	KNN	LR	RFC	Z-L	Z-R	Z-S	Z-X	Z-D
NR-AR	0.52	0.62	^a^0.59	0.4	0.56	0.50	0.62	0.43	0.60	^b^0.68
NR-AR-LBD	0.57	0.63	0.61	0.48	^a^0.62	0.60	0.71	0.60	^b^0.73	0.72
NR-AhR	0.44	0.47	^a^0.45	0.44	0.43	0.52	^b^0.61	0.47	0.54	0.59
NR-Aromatase	0.29	0.35	^a^0.32	0.25	0.29	0.28	^b^0.52	0.32	0.50	0.48
NR-ER	0.29	0.34	^a^0.33	0.24	0.29	0.37	0.42	0.32	0.40	^b^0.44
NR-ER-LBD	0.35	0.47	0.37	0.26	^a^0.42	0.45	0.56	0.36	^b^0.59	0.58
NR-PPAR-gamma	0.18	0.26	0.14	0.18	^a^0.22	0.32	0.50	0.30	^b^0.52	0.47
SR-ARE	0.28	0.36	0.25	^a^0.31	0.29	0.46	0.49	0.36	0.46	0.48
SR-ATAD5	0.24	0.26	^a^0.25	0.22	0.24	0.37	^b^0.59	0.36	0.53	0.55
SR-HSE	0.18	0.25	0.15	0.18	^a^0.20	0.31	^b^0.37	0.21	0.40	^b^0.37
SR-MMP	0.44	0.47	0.44	^a^0.47	0.43	0.63	^b^0.65	0.54	0.64	0.63
SR-p53	0.22	0.26	0.21	^a^0.24	0.23	0.42	^b^0.57	0.37	0.52	0.55

**Table 2:** Comparison of external and internal embeddings for PCA, UMAP, and VAE. Each cell represents the mean MCC score across nine different machine learning models (three random states × three classifiers). Values marked with an asterisk (*) highlight cases where, on average, models trained using external knowledge outperformed models trained on internal knowledge. Additionally, results marked with a quotation mark (‘) highlight cases where using external or internal knowledge yielded equal results (when rounded off to two decimal places).

Label	PCA		UMAP		VAE	
	IN	EX	IN	EX	IN	EX
NR-AR	0.45	0.43	‘ 0.47	‘ 0.47	0.45	0.44
NR-AR-LBD	0.45	0.43	* 0.45	* 0.53	‘ 0.43	‘ 0.43
NR-AhR	‘ 0.33	‘ 0.33	* 0.34	* 0.35	‘ 0.34	‘ 0.34
NR-Aromatase	0.22	0.18	0.18	0.15	‘ 0.21	‘ 0.21
NR-ER	0.22	0.21	‘ 0.23	‘ 0.23	‘ 0.27	0.24
NR-ER-LBD	0.31	0.26	‘ 0.26	‘ 0.26	‘ 0.28	‘ 0.28
NR-PPAR-gamma	‘ 0.14	‘ 0.14	* 0.09	* 0.11	0.11	0.09
SR-ARE	* 0.19	* 0.21	* 0.19	* 0.2	* 0.19	* 0.2
SR-ATAD5	0.16	0.13	* 0.12	* 0.16	* 0.14	* 0.15
SR-HSE	* 0.09	* 0.11	‘ 0.08	‘ 0.08	* 0.07	* 0.1
SR-MMP	‘ 0.36	‘ 0.36	‘ 0.32	‘ 0.32	* 0.35	* 0.36
SR-p53	0.19	0.18	0.16	0.14	* 0.15	* 0.17

**Table 3:** Classification results across all data sets and labels expressed by maximum values of the MCC. The fingerprint-based model maxima (FPR-BL) were set as 100%, while the embedding models referred to these 100%. Results assigned with an asterisk (*) outperformed baseline.

Label (endpoint)	FPR-BL	PCA-EX	PCA-IN	UMAP-EX	UMAP-IN	VAE-EX	VAE-IN
NR-AR	100	95	99	96	96	97	* 100
NR-AR-LBD	100	92	98	* 100	97	98	* 102
NR-AhR	100	84	85	90	86	83	85
NR-Aromatase	100	65	82	75	74	84	75
NR-ER	100	83	85	86	95	* 103	* 101
NR-ER-LBD	100	70	90	79	88	90	83
NR-PPAR-gamma	100	81	82	68	81	78	75
SR-ARE	100	74	69	70	69	63	63
SR-ATAD5	100	63	99	92	84	75	88
SR-HSE	100	82	77	56	90	65	55
SR-MMP	100	92	87	83	83	93	89
SR-p53	100	99	95	79	87	85	93

**Table 4:** Correlation of average classification results of the embedded classifiers (PCA-EX, UMAP-EX. VAE-EX) with the imbalance ratio (Pos class %) and baseline fingerprints classifiers (FPR-BL) with their respective silhouette coefficients—s(PCA), s(UMAP), and s(VAE).

	PCA-EX	UMAP-EX	VAE-EX
s(PCA)	0.74		
s(UMAP)		0.86	
s(VAE)			0.85
Pos class %	0.11	0.02	0.13
FPR-BL	0.98	0.98	0.99

**Table 5:** Elements of the confusion matrix that show the possible outcomes when predicting labels in Tox21.

Experimental/Model	Positive (Model) (1)	Negative (Model) (0)
Positive (Experimental) (1)	TP (experimentally active and predicted active)	FN (experimentally active, but predicted as inactive)
Negative (Experimental) (0)	FP (experimentally inactive, but predicted as active)	TN (inactive experimentally and predicted)

**Table 6:** Experimental matrix. See abbreviations below the table.

Predictive Variables	Classifier	Seed	Embedder	Emb. Dim.	CS1 Data Size	N Models
**Fingerprints (raw data)**	RFC, KNN, LR	1–3	N/A	N/A	N/A	144
**Internal emb.**	RFC, KNN, LR	1–3	PCA, UMAP, VAE	2–15	N/A	9072
**External emb.**	RFC, KNN, LR	1–3	PCA, UMAP, VAE	2–15	200–30,000	9072

CS1—compound set 1, Seed—random state in machine learning, RFC—random forests classifier, KNN—k-nearest neighbours classifier, LR—logistic regression, emb—Embeddings.

## Data Availability

The datasets were retrieved from https://tripod.nih.gov/tox21/assays/index.html (compound set 2) and https://zenodo.org/record/4248826 (compound set 1).
